# Reclassification Scheme for Image Analysis in GRASS GIS Using Gradient Boosting Algorithm: A Case of Djibouti, East Africa

**DOI:** 10.3390/jimaging11080249

**Published:** 2025-07-23

**Authors:** Polina Lemenkova

**Affiliations:** Department of Biological, Geological and Environmental Sciences, Alma Mater Studiorum-Università di Bologna, Via Irnerio 42, Emilia-Romagna, 40126 Bologna, Italy; polina.lemenkova2@unibo.it; Tel.: +39-3446928732

**Keywords:** image processing, image analysis, remote sensing, satellite image, geoinformatics, machine learning

## Abstract

Image analysis is a valuable approach in a wide array of environmental applications. Mapping land cover categories depicted from satellite images enables the monitoring of landscape dynamics. Such a technique plays a key role for land management and predictive ecosystem modelling. Satellite-based mapping of environmental dynamics enables us to define factors that trigger these processes and are crucial for our understanding of Earth system processes. In this study, a reclassification scheme of image analysis was developed for mapping the adjusted categorisation of land cover types using multispectral remote sensing datasets and Geographic Resources Analysis Support System (GRASS) Geographic Information System (GIS) software. The data included four Landsat 8–9 satellite images on 2015, 2019, 2021 and 2023. The sequence of time series was used to determine land cover dynamics. The classification scheme consisting of 17 initial land cover classes was employed by logical workflow to extract 10 key land cover types of the coastal areas of Bab-el-Mandeb Strait, southern Red Sea. Special attention is placed to identify changes in the land categories regarding the thermal saline lake, Lake Assal, with fluctuating salinity and water levels. The methodology included the use of machine learning (ML) image analysis GRASS GIS modules ‘r.reclass’ for the reclassification of a raster map based on category values. Other modules included ‘r.random’, ‘r.learn.train’ and ‘r.learn.predict’ for gradient boosting ML classifier and ‘i.cluster’ and ‘i.maxlik’ for clustering and maximum-likelihood discriminant analysis. To reveal changes in the land cover categories around the Lake of Assal, this study uses ML and reclassification methods for image analysis. Auxiliary modules included ‘i.group’, ‘r.import’ and other GRASS GIS scripting techniques applied to Landsat image processing and for the identification of land cover variables. The results of image processing demonstrated annual fluctuations in the landscapes around the saline lake and changes in semi-arid and desert land cover types over Djibouti. The increase in the extent of semi-desert areas and the decrease in natural vegetation proved the processes of desertification of the arid environment in Djibouti caused by climate effects. The developed land cover maps provided information for assessing spatial–temporal changes in Djibouti. The proposed ML-based methodology using GRASS GIS can be employed for integrating techniques of image analysis for land management in other arid regions of Africa.

## 1. Introduction

### 1.1. Background

Satellite images play a pivotal role in environmental monitoring for reconstructing the the history of landscapes on Earth. Understanding how land cover types change over time gives insight into the development of human–environment interactions [1,2,3]. The main threat of environmental changes are linked to climate issues such as desertification, erosion, landscape fragmentation, coastal degradation and deforestation. Most of this information can be discovered on spaceborne images due to the fundamental principle of remote sensing (RS)—differences in spectral reflectance of pixels corresponding to diverse objects on the Earth. Using such benefits of RS, many environmental processes and phenomena can be recognised on satellite images and Earth Observation (EO) data, including, for instance, deforestation, soil erosion and desertification.

The important application of EO data for environmental monitoring consists of time series analysis. Such a technique is based on the comparison of changes visible in several multi-temporal images to evaluate trends in vegetation coverage during a given temporal period [4,5]. This enables the detection of land cover changes and the identification of landscape dynamics [6,7]. EO data processing requires advanced techniques that allow us to find optimal and time-efficient solutions for solving complex geospatial tasks in image analysis. While traditional methods of Geographical Information Systems (GISs) have limitations, programming algorithms have significant benefits in the time series analysis of satellite images.

Technologies of image analysis play an essential role in the development of environmental monitoring. Traditional tools for processing RS data rely on GISs [8,9]. However, due to the large volume of RS data, the automation of image analysis is needed to effectively perform time series analysis and extract geoinformation. Recent technological developments have enabled novel methods for extracting geoinformation from spaceborne data, including image enhancement [10], image differentiation, rationing or principal component analysis [11]. Machine learning (ML) methods have been found to improve the efficiency of image analysis through automated algorithms. Existing cases of ML algorithms in cartography and RS tend to be related to problems of image processing that are similar to those faced by decision algorithms. In each case, ML algorithms aim to identify the best solution strategy for image classification [12].

Examples of ML algorithms include Random Forest (RF) [13,14], Support Vector Machines (SVMs) [15] and Convolutional Neural Networks (CNNs) [16]. Such approaches are derived from programming and are adapted to image analysis [17,18]. Examples of novel techniques in image analysis include heuristic clustering [19,20,21,22], flexible control of variables [23], optimised pattern recognition [24,25], ensemble learning [26] and objective analysis [27,28].

Following such applications of ML in Earth and climate sciences, this study reports the case of a novel independent reclassification scheme for image analysis at the pixel level using ML coupled with Geographic Resources Analysis Support System (GRASS) GIS and gradient boosting algorithm. Its advantages include the optimisation approach, which considers previous iterative cycles of image classification for the generation of new models and the narrowing of pixel assignment options to various land cover classes.

### 1.2. Objectives and Goals

The main goal is to identify changes in land cover types of Djibouti using the time series of satellite images by the reclassification scheme. GRASS GIS version 8.4.1 is an advanced scripting cartographic and RS software with a powerful computational engine for geospatial processing [29]. Developed by the international GRASS developer community [30], it is used for processing cartographic data, maps, models and satellite image analysis across various applications in geoinformatics. Some of its important benefits are a built-in framework and a Python Application Programming Interface (API), which supports the automation of data processing through scripting. To achieve this goal, the objective is to map land cover changes in the coastal region of Djibouti using RS data. The scripts are included and commented for reference. The specific objectives of this research are as follows:

1. To produce land cover maps based on the classification of Landsat satellite images from 2015, 2019, 2021 and 2023;
2. To quantify and visualise dynamic changes in land cover types for the identification of landscape dynamics;
3. To assess the accuracy of classification for vegetation mapping in central and coastal zones around Lake Assal.

The rest of this manuscript is organised as follows. Section 1 presents the introduction with a related literature review and identifies the objectives and goals. Section 2 describes the study area, focusing on environmental and climate–hydrological settings that influence landscape types. Section 3 introduces the materials and numerical methods used in this study, including the dataset and workflow, which are described as the methodological approach. Major technical procedures are described in the subsections that follow. In Section 4, we present and discuss the results with cases of land cover changes, comment on uncertainties, and provide and interpret a series of maps. Finally, Section 5 concludes the manuscript, summarising the findings and providing recommendations for future studies.

## 2. Study Area

**Figure 1:**
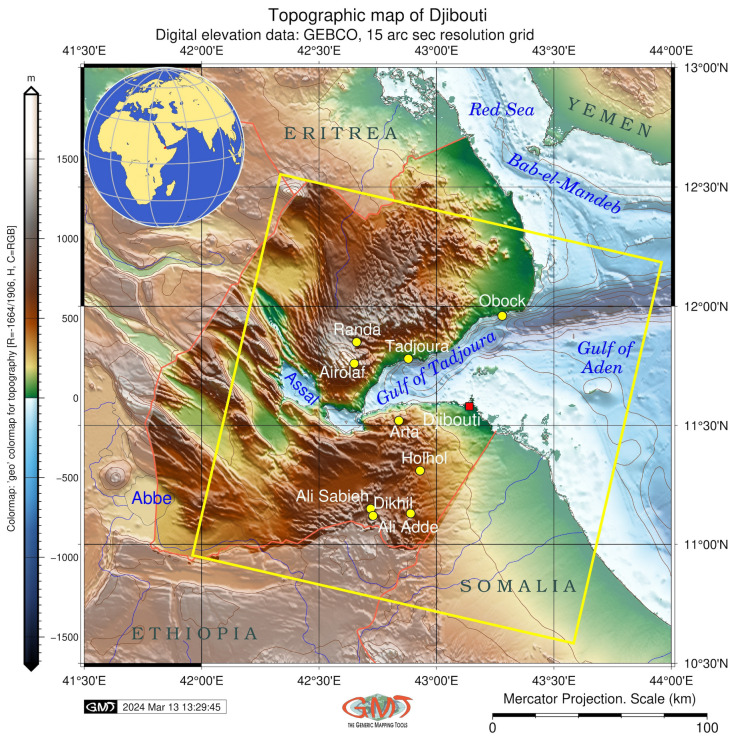
Topographic map of Djibouti with indicated study area (yellow rotated square) which shows the location of the Landsat images. Mapping software: Generic Mapping Tools (GMT) version 6.4.0. Data source: General Bathymetric Chart of the Oceans (GEBCO). Map source: Author.

This study area is located in Djibouti and surrounding coastal areas in the Gulf of Tadjoura, Bab-el-Mandeb Strait (Figure 1). Situated in the region of the Horn of Africa and surrounded by waters of the southern Red Sea, Djibouti is a strategic point with geo-maritime importance [31,32]. Djibouti is one of the hottest and driest places on Earth with many issues related to climate and weather processes [33]. The thermal crater saline lake, Lake Assal, is located in this area, which is the lowest point on land in East Africa with extreme levels of evaporation. The topography affects temperature and precipitation, which vary according to the elevation and differ in coastal and mountainous regions [34].

This region experiences fluctuations in salinity and water levels due to seasonal and annual changes in temperature and precipitation. Dominating landscapes are represented by dry mountain vegetation typical of arid climates. The distribution and genera of flora and fauna are controlled by low precipitation and high temperatures in semi-desert and desert environments [35,36,37]. For example, the 2011 drought in East Africa affected rain-fed agriculture in Djibouti, which led to a regional famine [38,39,40]. Drought-induced crises endanger long-term investments and development, with catastrophic human costs and effects on vulnerable regions of the country [41,42,43].

The drought-prone regions of Djibouti have experienced strong effects from climate change, including fluctuations in the hydrological regime of thermal lakes. The arid climate has triggered environmental problems; for example, it has desiccated and abandoned agricultural lands, and salinated and blocked water sources due to sea level rise and flash floods [44,45]. Soil erosion and land degradation vary across topographically diverse landscapes, reflecting the prevailing climatic conditions. [46,47]. Moreover, environmental pollution has been reported along the coasts of the Gulf of Tadjoura [48,49].

**Figure 2:**
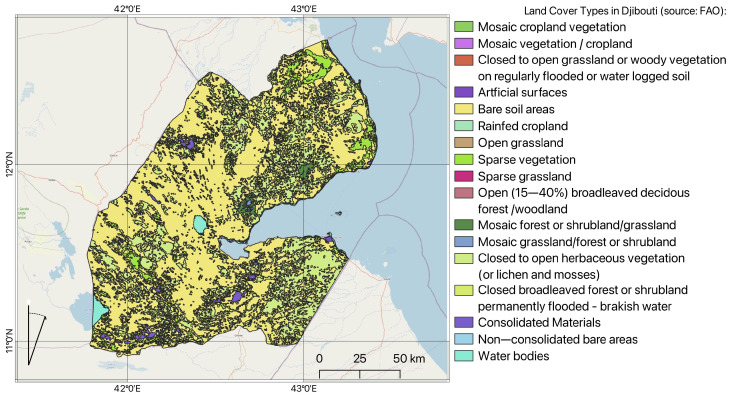
Map of land cover types over Djibouti, showing the distribution of major vegetation and land cover types. Software: Quantum Geographic Information System (QGIS). Data source: Food and Agriculture Organisation (FAO). Map source: Author.

The surroundings of the Horn of Africa are notable for climate–hydrological fluctuations which cause land cover changes [50,51]. The coastal region located in the Bab-el-Mandeb region includes diverse types of xeric grasslands and shrubland typical for arid ecoregions (Figure 2). This region consists of various land cover classes typical for semi-desert areas: dry shrub steppe and grassland, including species of *Vachellia tortilis*, *Vachellia nubica*, and *Balanites aegyptiaca*. Many scarce vegetation spots include scattered trees or shrubs on the sandy plains of bare lands and semi-deserts.

The eastern strip along the Red Sea coast is part of the Eritrean coastal desert [52]. It includes vegetation along the muddy wadi mouths in diverse habitats [53], grasslands [54,55], and secondary growth areas in the urban areas. The floodplains along the wadi rivers include scarce vegetation typical for semi-desert ecosystems. Xeric vegetation, farmland, mangroves along the coasts of the Red Sea and valleys of wadi [56,57], coral reefs along the coasts [58,59,60] and urban areas collectively make up the region’s landscape. Djibouti is one of the most seismically unstable zones of Africa with high tectonic–magmatic activities [61,62,63,64] due to the closeness of the Afar Triple Junction [65,66,67]. The thermal regime around the Red Sea is explained by its structural connection to the active zone of the East African Rift [68,69,70,71,72].

Such complex geological setting and arid environment have resulted in the deficit of water and favoured the distribution of thermal springs with high salinity, located in three regions—Lake Assal, Lake Hanle and Lake Abhe [73,74]. The craton origin of Lake Assal is related to the Afar Triple Junction [75,76,77]. The geochemical composition of the Assal Rift includes evaporitic halite deposits [78,79]. Its location in the arid zone with strong evaporation and no riverine input contributed to the lake’s increase in salinity, with a high brine concentration above 100 g/L [80]. Fluctuations of this concentration are related to evaporation, the hot climate and geothermal processes [81].

## 3. Materials and Methods

Our methodology presents a case of image reclassification and analysis using ML techniques for environmental mapping in Djibouti, East Africa. A classification scheme consisting of 17 initial land cover classes was employed through a logical sequence of GRASS GIS modules to extract land cover information of the study area in Djibouti. Reclassification includes the sequence of changing the values in a raster dataset to new values. The process is based on a set of rules which defined the criteria of updates in classes. This operation in GRASS GIS was applied for the simplification of land cover polygons, standardisation of assignment of pixels to classes and preparation of data for land cover analysis.

### 3.1. Data

**Figure 3:**
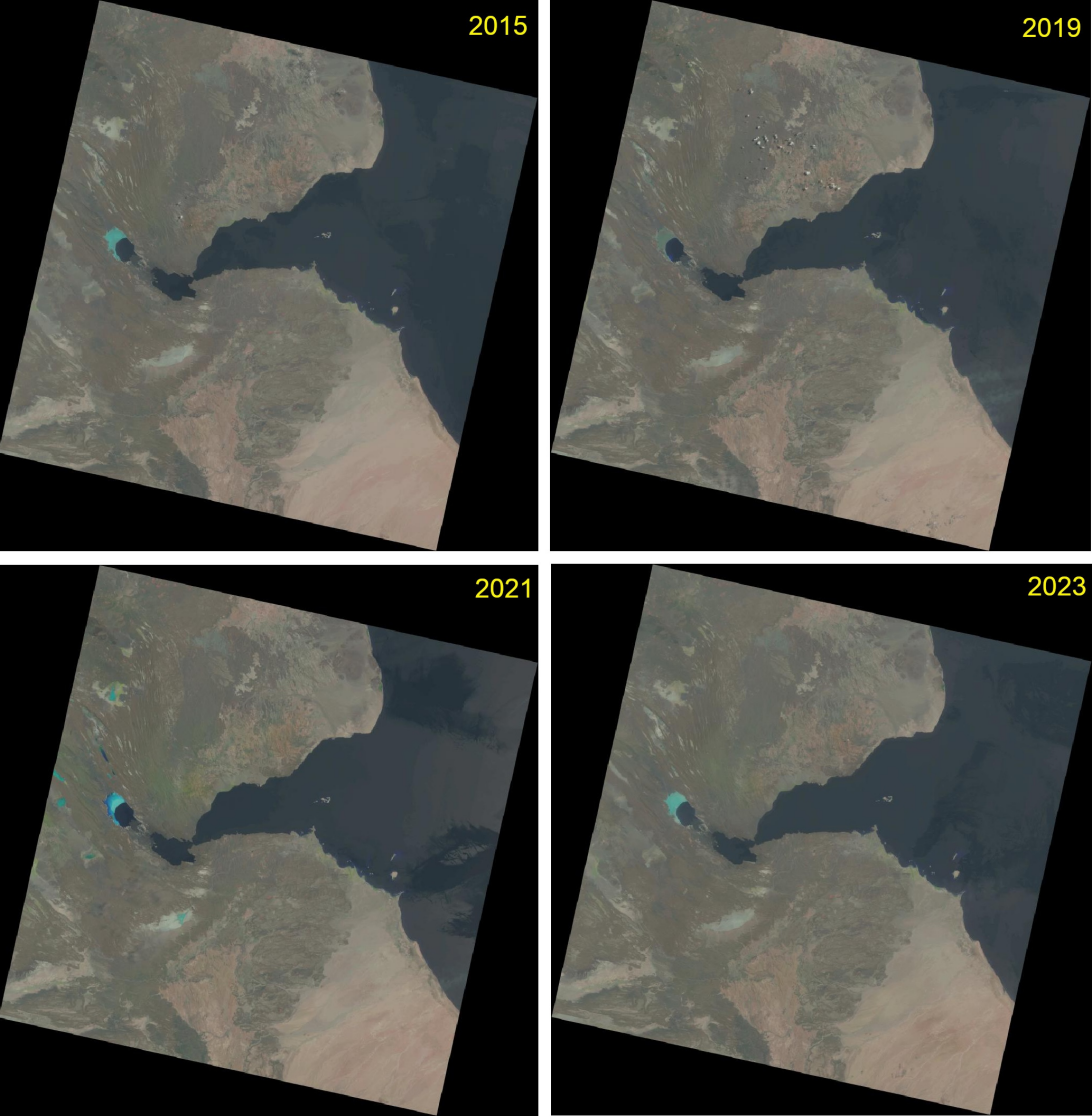
Original Landsat 8–9 OLI/TIRS scenes of Djibouti from 2015, 2019, 2021 and 2023 used for image processing. Image source: United States Geological Survey (USGS). Compilation: Author.

The data used in this study include four images from the Landsat 8–9 Operational Land Imager (OLI) and Thermal Infrared Sensor (TIRS): the training image from 2015, and images with biannual intervals from 2019, 2021 and 2023 (Figure 3).

The Landsat images were selected as the data source due to their quality and reliability [82,83,84]. Earlier studies reported the effective use of the RS data in environmental studies which prove their utility and value [85,86,87,88,89,90,91]. Nowadays, satellite images are excellent sources of geoinformation for mapping arid regions [12,92,93]. Spaceborne data are successfully used in the analysis of land cover change and deforestation in tropical forests, and for reporting landscape dynamics [94,95,96,97]. The importance of Landsat data for environmental research has been described earlier [98,99,100].

In this study, four Landsat images were taken on the following dates: (1) 2 July 2015; (2) 23 July 2017; (3) 19 August 2021; and (4) 16 July 2023. The metadata are summarised in Table 1. The cloudiness percentage is less than 10% for all the images. The technical parameters of the scenes are provided in Table 2. Common data for all four images are as follows: Datum and Ellipsoid: World Geodetic System 1984 (WGS84); Product Map Projection L1: Universal Transverse Mercator (UTM) zone 38 (for Djibouti); Worldwide Reference System (WRS): Path—166, Row—52; Landsat Collection Number: 2; Category: T1; Station Identifier: LGN; and Sensor Identifier: OLI/TIRS. The images were taken during day time in nadir with a roll angle of 1° for the 2015 image and 0° for the images from 2017, 2021 and 2023.

The image quality score was 9 for all scenes, according to the Landsat specifications. The remaining various metadata for each scene are summarised in Table 2. The geographic characteristics of the images are referenced in Table 3. The Landsat Scene Identifiers (IDs) are provided below.

The images have been filtered to reduce noise due to cloudiness and atmospheric effects. Images were systematically selected from the USGS EarthExplorer repository and taken at the end of the dry season. During this period, cloud cover is generally low but vegetation appears bright green and contrasts with all other land cover types.

### 3.2. Workflow

**Figure 4:**
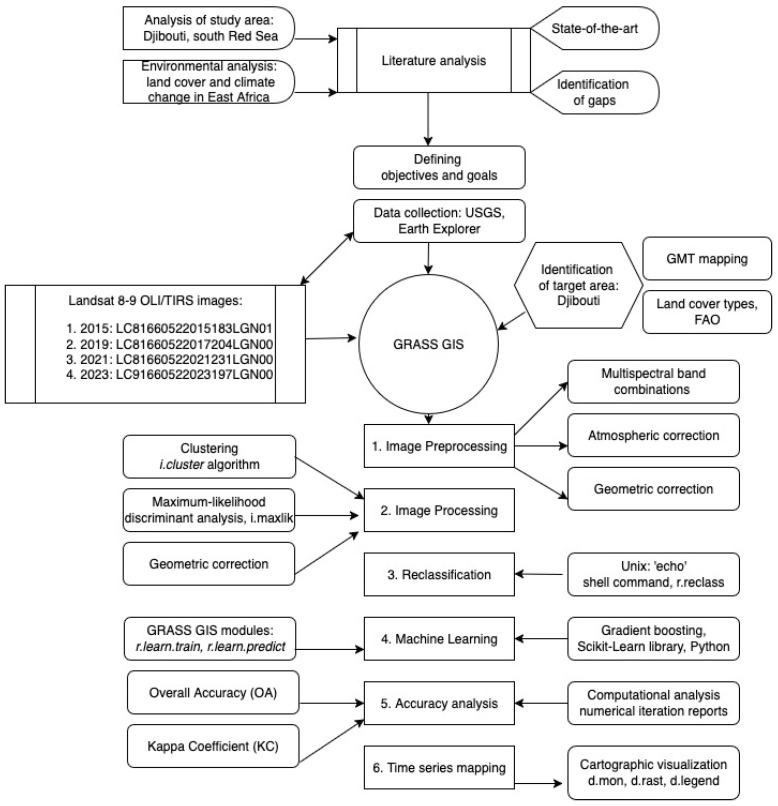
Workflow scheme. Source: Author.

The schematic workflow of the research is presented in Figure 4.

The image processing used to create a land cover map from Landsat OLI/TIRS imagery was carried out in six steps using the following techniques of GRASS GIS:

- Image import;
- Data exploration and analysis;
- Classification of images using MaxLike method;
- Reclassification scheme using ‘echo’ of Unix and ‘r.reclass’ module;
- Classification of images using gradient boosting algorithm of ML;
- Image post-processing and mapping;
- Accuracy assessment.

The workflow is based on scripting techniques of GRASS GIS for image analysis (software developed originally by the U.S. Army Construction Engineering Research Laboratories in 1982, now maintained by he developer team in Champaign, IL, USA). The topographic map is plotted using existing techniques in GMT version 6.4.0. [101,102,103,104,105] (software developed originally in 1988 by Paul Wessel and Walter H. F. Smith, in Lamont-Doherty Earth Observatory of Columbia University, Palisades, NY, USA).

### 3.3. Image Preprocessing

The Landsat 8–9 OLI/TIRS images were geometrically rectified according to EPSG coordinates: 2095, Universal Transverse Mercator (UTM) zone for Djibouti according to the USGS technical characteristics. In this study, a scripting method of GRASS GIS was applied, which uses several modules with code snippets explained and commented below.

The images were imported into the working folder using the ‘r.import’ module, then the same procedure was repeated for all the bands and the four images used in this study (2015, 2019, 2021 and 2023). Afterwards, the relief map was imported from GEBCO. The region was set up to the geospatial extent with the isolines obtained using the ‘r.contour’ module with a 200 m interval. To facilitate data processing, the best combinations of Landsat bands were stacked into coloured composites. The bands that produced the best separation for pixels were processed, excluding the panchromatic channels. The Tag Image File Format (TIFF) files were joined using the ‘i.group’ module and enhanced using preprocessing steps which included contrast stretching and atmospheric correction.

### 3.4. Clustering

The clustering was performed by the ‘i.cluster’ module of GRASS GIS, which generates a signature file and reports results using the k-means clustering algorithm, as explained in previous studies [106,107,108,109]. Signature file determines the categories (classes) of land cover types using the analysis of spectral reflectance of the pixels and contains clusters and covariance matrices for each image. The initial means for each class before data processing reported in Appendix A indicate the starting seed values of the multispectral band before iteration is assigned according to the signature files. The means are then calculated during the ‘i.cluster’ procedure and recalculated following the principles of maximal class separation (the contrast between the classes) and minimal class size (the details of classification with respect to a spatial resolution of 30 m). The table in Appendix A reports such values for each of the 10 classes and 7 multispectral bands of Landsat, starting from 1—‘Coastal aerosol’ to 7—‘SWIR-2’.

**Figure 5:**
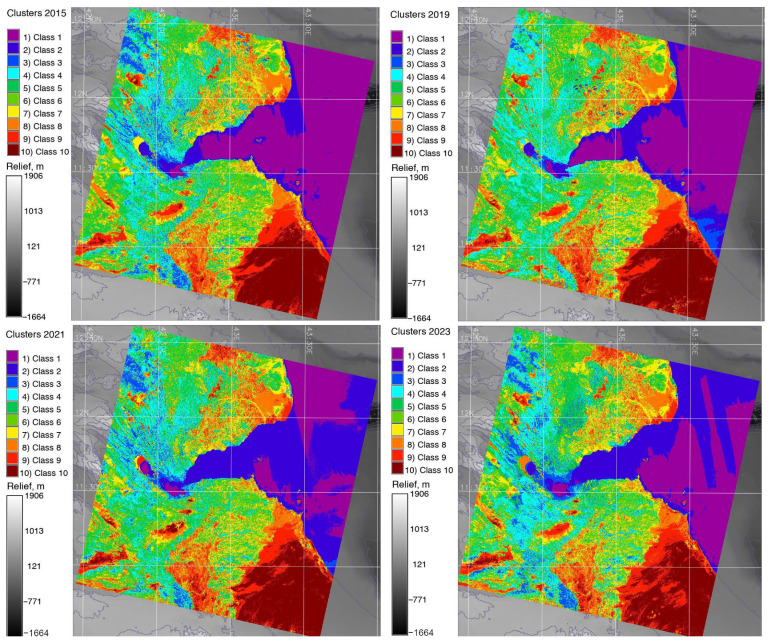
Maps of land cover types based on the classified Landsat 8–9 OLI/TIRS images from 2015, 2019, 2021 and 2023 covering Djibouti. The approach involved the maximum-likelihood discriminant analysis classifier. Background relief: GEBCO. Software: GRASS GIS. Mapping source: Author.

The initial mean values for each land cover class for the years 2015, 2017, 2021 and 2023 are reported in Table A1, Table A2, Table A3 and Table A4; see Appendix A. Afterwards, the assignment of pixels was performed iteratively for each land cover class. The same was applied for all images for the years 2015, 2017, 2021 and 2023 with a biannual gap. The classification was performed using the maximum-likelihood discriminant analysis classifier by the ‘i.maxlik’ module [110,111]. The algorithm used the signature file calculated in the previous step. Using the signature file, the land cover types were classified using maximum-likelihood discriminant analysis (Figure 5).

### 3.5. Accuracy Analysis

**Figure 6:**
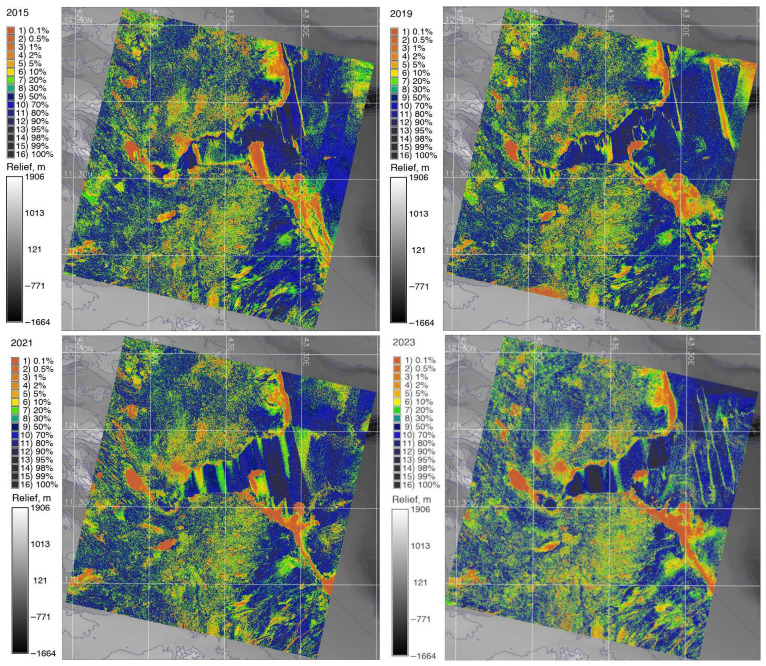
Accuracy analysis of image classification using reject threshold probability algorithm and the chi-squared test, which was applied to the reclassified images. Source: Author.

The analysis of accuracy (Figure 6) revealed the areas of misclassified pixels within water bodies, including the Bab-el-Mandeb Strait. The accuracy assessment, showing the probability of correctly assigning each pixel to its respective class across all images, is presented in Figure 6 and calculated with the averages of each calculated band. Afterwards, based on the classified ten categories in the images, two of them (‘water in the sea’ and ‘water in shelf’ areas) were merged as a single class ‘water’, and the names of the classes were assigned to each numerical cluster.

After this step, ML was applied to all the images. The ML step started with the generation of training pixels from land cover classification performed in 2015, which were used as seed data. The extraction of training data was conducted using the ‘r.random’ module. Afterwards, the training pixels were used for classification on the Landsat images (example for 2023) by training a model using the ‘r.learn.train’ module. The gradient boosting classifier was used as an algorithm to perform ML classification. Next, the prediction was performed using the ‘r.learn.predict’ ML module, which applied a fitted estimator from the Python’s Scikit-Learn library [112] to raster images in the imagery group. In the next step, the computed raster categories were automatically applied to the classification output and checked using the ‘r.category’ module.

### 3.6. Gradient Boosting

The images were processed using the gradient boosting ML method of image classification. The essential concept of this algorithm is setting the target outcomes of classified images for the next model based on previous iterations, which in the end minimises the error of classification. Gradient boosting presents an advanced ensemble technique of ML, which combines the predictions of multiple classification steps by the principle of decision trees. Such an approach presents a novel method of satellite image processing based on the supervisor approach. The algorithm classifies images using the iterative approach which considers the previous and next classification steps as loops. It combines several decision trees as simplified learners to create a strong prediction mode. By minimising a loss function embedded in the algorithm, each new learner fixes the mistakes committed by the group of prior learners. In this way, the accuracy and predictive capacity of the classifier are increased iteratively, which is directed by the gradient of the loss function.

The core idea is that the algorithm finds the best possible next model when combined with previous ones. In this way, it minimises the overall prediction error. The advantage of such an approach is that it improves the accuracy of classification through the convergence of decision cycles and assigns cells that constitute the mosaic of pixels on a raster image to the defined categories. When several images are processed as a time series, this enables us to effectively monitor landscape dynamics and detect changes.

The class separability matrices were computed for each pair of years (2015–2017 and 2021–2023) and show the distinguishability between classes (Equations (A1) and (A2)). The iterative cycles of image classification are summarised in Appendix B in four statistical tables for each of the study periods, respectively. They report the pixels’ distribution by categories of land cover classes for each year: 2015, 2017, 2021 and 2023. The values indicate percent convergence, which shows at which values the cluster means of land cover categories become stable during iterative classification. Hence, iterative processes aim to achieve the maximal percentage of pixels that no longer move from cluster to cluster during iteration and reach the best possible values.

### 3.7. Reclassification

To improve accuracy, the images were reclassified using the principle of reclassification scheme. When clusters are being generated by the algorithm, the means of classified categories constantly change, because cells are assigned to these classes and the mean is then recalculated to include new pixels. The goal of the iteration is to maximise the distances between the classes through recalculation. To this end, after the creation of all clusters and the assignment of pixels into these groups, the algorithm ‘i.cluster’ changes cluster means through iteration processes. In this way, the algorithm attempts to increase the contrast between the classes, i.e., the numerical gap in values between the classes.

### 3.8. Generalisation of FAO Land Cover Map

The FAO land cover map of Djibouti was generalised from the classes of the Land Cover Classification System (LCCS) according to a technical report on the country. The scheme covering land cover categories comprising 10 classes was used and adopted for the study areas within the country (Table 4). This part of the work was performed using QGIS software, version 3.42.2 ‘Münster’. The QGIS was initially developed by G. Sherman and now a project of the Open Source Geospatial Foundation.

The reclassified image was generated for each year using the ‘r.reclass’ module of GRASS GIS and ‘echo’ Unix command. The raster map was reclassified based on the category values, and new raster maps for the years 2015, 2019, 2021 and 2023 were generated. The category values of the reclassified maps are based on an iteration of the categories using the information generated in ‘landusereclass.txt’ file. These categories were controlled using the ‘r.category’ module. The maps were visualised using modules ‘d.mon’ and ‘g.region’. The color palette was selected from the color tables. Afterwards, the maps were displayed using a combination of ‘d.rast’ and ‘d.vect’ modules. Finally, a cartographic grid was added along with the legends by the ‘d.legend’ module.

The reclassification was performed since water surfaces in the Bab-el-Mandeb Strait and southern Red Sea had different colours. Nevertheless, they belong to the same class of water. The differences in the coastal regions were smooth at the time of image acquisition and appear as dark colours in all images. With each iteration, the values of the means shift to a higher percentage of pixels within each cluster. This is illustrated in the class separability matrix, which shows the distinction between the categories (Equations (A1) and (A2); Appendix C). As means never become completely static, a % convergence and a maximum number of iterations are defined to finalise the process of reclassification before the maximum number of cycles is reached. The final values of the computed class means for each land category are reported in Appendix D. Once the maximum number of reclassification cycles is reached, the optimal % convergence is achieved through the increased number of iterations during the ‘i.cluster’ procedure. Here, the number of cycles depends on the complexity of the terrain and land cover patterns.

## 4. Results and Discussion

### 4.1. Land Cover Change Analysis

The results of the classified satellite images are presented in Figure 5. The study area was categorised into 10 distinct land cover classes following the FAO classification scheme: (1) salt plains and hardpans; (2) barren land; (3) water bodies; (4) mangroves and aquatic vegetation on regularly flooded (temporarily or permanently) fresh or brackish water; (5) bushes and shrubs; (6) farmland, shrubs and irrigated trees; (7) built-up areas, artificial surfaces and associated areas; (8) broadleaved semi-deciduous forest and woodland; (9) mosaic cropland in cultivated areas; and (10) sparse vegetation in desert areas. Among these classes, the following categories included sub-clusters: water bodies comprise ponds, areas covered by marshes, rivers, estuaries, and coastal waterways; bushland and shrubland include non-classified types of vegetation such as grasslands, shrubs, rangelands, and savannah; and coastal areas surrounding Lake Assal are categorised by salt-tolerant vegetation and mangroves.

Over an estimated period of a decade, from 2013 to 2023, image analysis revealed certain changes in land cover types across the study area of Djibouti. Thus, it is noteworthy that the amount of mangroves along the coastal areas decreased slightly, shrinking by 0.35 km^2^ from 6.28 km^2^ to 5.93 km^2^ during this period. Furthermore, a large area of 1392 km^2^ that had previously been covered by bushes and shrubland was converted into other land use categories, resulting in a decrease in coverage from 1194 km^2^. As a result of this reduction, the percentage of bush cover decreased to 15%. Over the course of the studied period, both salt-covered areas around Lake Assal and croplands showed downward tendencies, with farmland areas falling from 3.21 km^2^ to 2.75 km^2^ and salt coverage from 17 km^2^ to 16.04 km^2^.

Additionally, saline waters mixed with the fresh waters of the estuaries of the Gulf of Tadjoura have a lighter coloration than those of the open sea. Vegetation types such as mosaic croplands, sparse vegetation, xeric shrubland and grassland or bare soil areas are detected and indicated on the maps. Broadleaved deciduous vegetation is relatively scarce in the study area. The regions of shrub, and flooded brackish water areas are mostly occupied by mangrove forests, which have stronger spectral signals and appear as various shades of colors. The computational results of the pixels by land cover classes are summarised in Table 5.

Xeric shrubland and sparse deciduous forests typical in Djibouti are located along the coasts of the Red Sea or Gulf of Tadjoura. Spectral signatures of this class differ from those of mosaic vegetation (semi-deciduous forests or sparse lands) found in inland regions of the country, on the border with Ethiopia and Eritrea. Thin coastal regions covered with mangrove plants along the coasts of the Bab-el-Mandeb Strait are identified as wetlands partially submerged in water. This type of vegetation shows little difference compared to the salt areas of Lake Assal, which experience significant changes.

There were also notable expansions in other land cover categories of landscapes in Djibouti. For example, the area of bare lands and in the interior deserts (western region of the country) increased significantly between the estimated period of 2015 and 2023. Specifically, the areas expanded from 5.56 km^2^ to 6.24 km^2^. This change highlights the processes of desertification of regional landscapes, which is caused by recent climate change and global warming. Hence, desertification processes have become notable over time, with arid regions occupying more areas in inner regions of the country.

### 4.2. Uncertainties and Sources of Error

Understanding possible uncertainties underlying the classification schemes in RS data processing is imperative. Accurate vegetation mapping using satellite image classification is not easy to achieve due to the spectral confusion between different vegetation types and similarity in spectral reflectance of various plant species [113,114,115,116]. Therefore, accuracy assessment is a crucial step in the evaluation process. In this case, the generated map was compared to the ground truth values, represented in this case by sample points taken from the FAO-based map. The overall accuracy (OA) and Kappa coefficient (KC) were computed to evaluate the classified map’s accuracy by measuring the similarity between the sample points and the correctly and incorrectly categorised classes (Table 6).

The sources of errors in automatic classification are also caused by the individual patterns of land cover categories on Earth; the data are not normally distributed in most cases. Moreover, the light signature of the deciduous broadleaved plants enabled us to detect occasionally growing plants on the mountainous slopes in the central regions of the country where precipitation is higher than in semi-desert areas. Thus, deciduous shrubland in all the images is attributed to the high backscatter coefficients of this vegetation type and spectral reflection effects. Therefore, validation using the computed KC was performed and is reported in Table 6.

In order to avoid uncertainties in classification caused by such cases, coastal types of vegetation were grouped in the same class with other plants having similar spectral properties (grassland). The classification accuracy was performed in order to evaluate possible sources of errors. To this end, images were processed using a rejection probability test, which examines the correctness of the pixel’s classification using the chi-squared test. This made it possible to evaluate the classification of different types of land cover types over the study area using a traditional approach of GRASS GIS based on automated clustering (Figure 6).

### 4.3. Interpretation and Data Analysis

**Figure 7:**
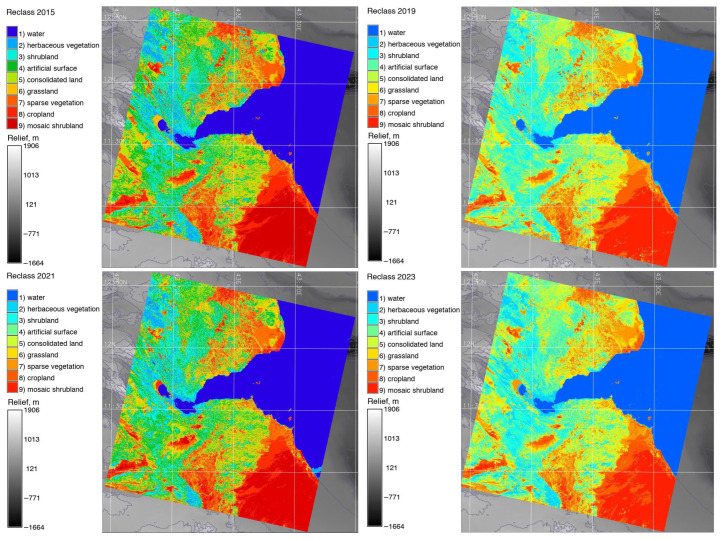
Reclassified satellite images after reclassification used by the ‘r.reclass’ technique. Images are from 2019, 2021 and 2023 covering Djibouti. Background: GEBCO. Software: GRASS GIS. Mapping source: Author.

**Figure 8:**
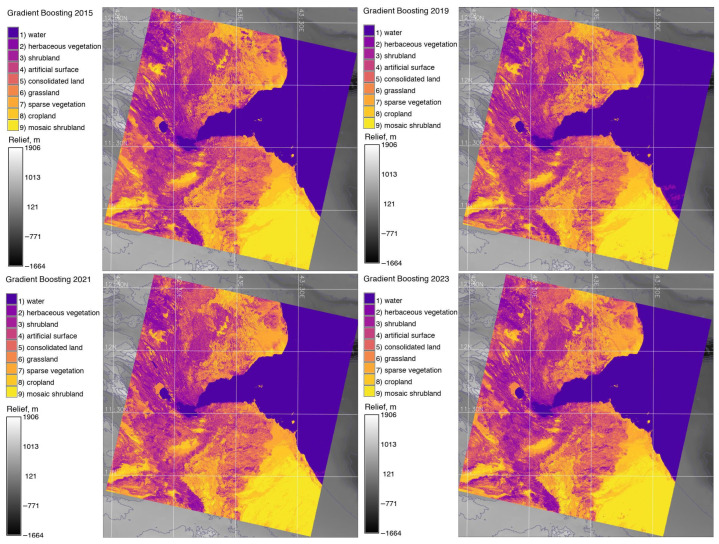
Image classification based on the ML gradient boosting classifier algorithm applied for Landsat scenes covering Djibouti in 2015, 2019, 2021 and 2023. Background relief: GEBCO. Software: GRASS GIS. Mapping source: Author.

Major land cover types in Djibouti include xeric shrubland, bare land areas in mountainous deserts, estuary of the Gulf of Tadjoura, cropland areas and sparse vegetation. Multispectral bands of the images were analysed and compared using a spectral diagram and spatial analysis. The main land cover maps of Djibouti generated using reclassified scenes and ML-based analysis for the images is displayed in Figure 7 and Figure 8.

The reclassified images are shown in Figure 7. Here, the areas of water are merged into one class, which improved the classification. Nevertheless, the neighbouring regions containing vegetation types with similar pixel reflectance were misclassified, which required the ML approach for image processing. A fluctuation in Lake Assal visible on the images is related to climate effects and geothermal activities, as is also mentioned in previous studies. This study revealed that similar to the fluctuations of saline sabkhas in the northern Sahara [117,118], the saline lake, Lake Assal, also witnessed slight changes in the water surface and extent of the lacustrine coasts. Nevertheless, the nature of such physiographic variations is more related to the geologic origin [119].

Landscape change detection was performed through a comparison among the land cover types detected on the ML-based classified satellite images obtained on different dates. The classification of the images is based on an automated evaluation of the spectral reflectance values of the individual pixels on the images, which are assigned to different land cover classes. The comparative analysis of the classified images enabled us to detect landscape changes. The observed transitions of categories were the loss of intact vegetation areas; fluctuations in the saline lake, Lake Assal; gains in vegetation and coastal areas; and secondary vegetation growth. These transitions correspond to the predominant patterns of vegetation response to climate warming and growth annual temperatures, which are visible when comparing central and coastal regions of Djibouti, where the latter have been influenced by the marine climate, especially in the coastal areas of the Gulf of Tadjoura.

The traditional (maximal likelihood) and ML classification maps were compared for 2015, 2019, 2021 and 2023. Comparing the changes on classified images from different dates shows the dynamics of the landscapes over Djibouti from 2015 until 2023. The differences between the land cover or land use types are presented in Figure 5, Figure 7 and Figure 8. Figure 5 shows the results of the classification made using the maximal likelihood approach; Figure 6 shows the accuracy assessment; Figure 7 shows the reclassified maps; and Figure 8 shows the results of the classification performed using the ML approach.

The comparison of the classified maps revealed that natural vegetation, such as herbaceous and mosaic shrubland, cropland and grassland, has decreased while the sparse vegetation and bare soil areas typical of desert and semi-desert areas in Djibouti have increased. Changes in land cover types in the coastal region of Bab-el-Mandeb and central Djibouti were detected and visualised in the target landscapes over the period from 2015 to 2023. The images were compared based on different dates to assess the landscape dynamics, which is defined by the evaluation of land cover types through the identification of various land cover patches, in different regions of Djibouti.

## 5. Conclusions

This paper demonstrated the efficiency of RS data processing using ML methods for visualising land cover changes. Using this approach, the dynamics of land cover types has been detected by identifying differences in the spectral reflectance of pixels on images assigned to different categories by ML algorithms of gradient boosting. This method was tested, and has been explained and presented in the form of a series of new maps covering Djibouti. Interdisciplinary problems in geographical sciences often require decisions to be made by diversified approaches. ML algorithms, such as the gradient boosting classifier, improved mapping workflow through programming to optimise RS data classification. When supplementary modules of GIS are used, such tools point out the shortcomings of traditional methods in geoinformatics. The development of cartographic tools for RS data processing using ML is a challenging task. As technologies mature along with the development of programming algorithms, cartographic modelling and the analysis of landscape changes improve by integrating applications of scripting methods. Here, we have demonstrated the use of such tools using Python’s Scikit-Learn library, adapted to GRASS GIS.

Scripting cartographic tools enable us to highlight salient aspects of environmental dynamics through the automation of mapping and data visualisation. They support geospatial data processing, modelling, visualisation and interpretation. For such geologically complex areas, integrated programming methods assist in handling the issue of data processing in a spatially expressive manner. The effectiveness of ML is explained through the advanced algorithms of image classification and automated workflow. Hence, the use of ML tools is essential for implementing the workflow of processing geographic data to reveal and visualise environmental problems. Their applications demonstrate a prominent role of scripting for geospatial data handling and environmental analysis. As ML algorithms are developed and implemented in RS and mapping, their performance supports the computational complexity of the tasks involved in cartographic data processing. Many case studies on geographical analysis and mapping, which use conventional GISs, for example, require highly computational and costly mapping workload due to diversified cartographic tasks. In such cases, the use of ML, most notably gradient boosting, is an essential solution to optimising cartographic workflow.

Future research can use the thematic maps of land cover types and continue with the analysis of neighbouring regions of the Bab-el-Mandeb Straight for environmental monitoring. Moreover, it can enhance the thematic and topical research of East Africa, particularly in relation to the geological exploration of Djibouti, environmental analysis, and geophysical monitoring of tectonically active regions within the Afar Triple Junction. Furthermore, to continue this study, future works can adapt ML techniques of GRASS GIS for land cover monitoring of other regions over an extended period to analyse landscape dynamics using time series analysis in regions around the Red Sea, East Africa.

## Figures and Tables

**Table 1:** Identifiers (IDs) and acquisition dates of the multispectral Landsat 8–9 OLI/TIRS images.

Date	Landsat Product Identifier	Scene ID
2 July 2015	LC08_L2SP_166052_20150702_20200909_02_T1	LC81660522015183LGN01
23 July 2017	LC08_L2SP_166052_20170723_20200903_02_T1	LC81660522017204LGN00
19 August 2021	LC08_L2SP_166052_20210819_20210827_02_T1	LC81660522021231LGN00
16 July 2023	LC09_L2SP_166052_20230716_20230719_02_T1	LC91660522023197LGN00

**Table 2:** Metadata of the Landsat 8–9 OLI/TIRS satellite images. Source: USGS (EarthExplorer).

Dataset Attribute	Attribute Value	Attribute Value	Attribute Value	Attribute Value
Date Acquired	16 July 2023	19 August 2021	23 July 2017	2 July 2015
Date Product Generated L2	19 July 2023	27 August 2021	3 September 2020	9 September 2020
Date Product Generated L1	16 July 2023	27 August 2021	3 September 2020	9 September 2020
Land Cloud Cover	0.02	0.19	0.45	0.08
Scene Cloud Cover L1	0.06	0.71	12.03	0.08
Ground Control Points Model	752	722	742	768
Ground Control Points Version	5	5	5	5
Geometric RMSE Model	3563	2963	2977	2098
Geometric RMSE Model X	2240	2009	1967	1089
Geometric RMSE Model Y	2771	2179	2235	1794
Processing Software Version	LPGS_16.3.0	LPGS_15.5.0	LPGS_15.3.1c	LPGS_15.3.1c
Sun Elevation L0RA	62.48488638	64.48715625	62.85521856	62.40770766
Sun Azimuth L0RA	65.62042782	84.79256539	68.51197578	61.89003236
TIRS SSM Model	N/A	FINAL	FINAL	ACTUAL
Scene Center Latitude	11.56786	11.56779	11.56783	11.56778
Scene Center Longitude	42.94139	42.97394	42.97879	42.96942
Corner Upper Left Latitude	12.60889	12.60924	12.60931	12.60921
Corner Upper Left Longitude	41.88103	41.91137	41.91688	41.90861
Corner Upper Right Latitude	12.62518	12.62530	12.62532	12.62529
Corner Upper Right Longitude	43.98441	44.01755	44.02308	44.01479
Corner Lower Left Latitude	10.50122	10.50151	10.50157	10.50149
Corner Lower Left Longitude	41.90427	41.93439	41.93986	41.93165
Corner Lower Right Latitude	10.51472	10.51483	10.51484	10.51482
Corner Lower Right Longitude	43.99199	44.02489	44.03037	44.02215

**Table 3:** Technical characteristics and geospatial location of the multispectral Landsat images.

N-S	Data Values	E-W	Data Values
North:	1,395,915.00	East:	393,015.00
South:	1,162,485.00	West:	164,085.00
Resolution:	30.00 m	Cells:	59,376,811
Rows:	7781	Columns:	7631

**Table 4:** Training pixels for validation under LCCS for Djibouti, derived from FAO classification scheme of land cover types: spatial distribution of the training ground truth samples.

ID	Landsat 8–9 OLI/TIRS Land Cover Class	FAO LCCS Codes
1	Salt Plains and Hardpans	6020
2	Barren Land	6001, 6004
3	Water Bodies	7001 // 8001
4	Mangroves and Aquatic Vegetation on Regularly Flooded (Temporarily or Permanently) Fresh or Brakish Water	41,653-R1,R2 // 41,739-R1 41,521-R1,R2 // 41,597-R1,R2
5	Bushes and Shrubs	11,490 // 11,494
6	Farmland, Shrubs and Irrigated Trees	11,499 // 11,500 // 30,001, 11,491, 11,495
7	Built-up Areas, Artificial Surfaces and Associated Areas	0010
8	Broadleaved Semi-deciduous Forest // Woodland	21,496 // 21,497–15,048 // 21,496–12,134 // 21,497-12,940
9	Mosaic Cropland (Cultivated Areas)	0003–0004
10	Sparse Vegetation	20,049, 20,056 20,058

Information source: FAO (land use statistics).

**Table 5:** Distribution of pixels by land cover classes: biannual variations in study area of Djibouti over 10 land cover classes in summer periods from 2015 to 2023.

Class	1	2	3	4	5	6	7	8	9	10
2015	1531	251	314	755	964	761	699	616	448	591
2017	1452	361	120	746	1031	813	672	714	502	609
2021	588	1225	223	699	1007	798	608	644	495	644
2023	842	915	367	733	965	840	672	627	533	547

**Table 6:** Error matrix for validation of gradient boosting ML algorithm across land cover categories.

Class	Salt Plains	Barren Land	Water	Mangrove	Shrub	Farmland	Built-Up Area	Forest	Cropland	Sparse Vegetation	Total
1	3503	12	4	7	31	4	29	2	9	21	3622
2	1	1651	43	4	5	0	8	12	0	2	1726
3	0	1	1018	21	0	8	15	4	2	12	1081
4	2	3	14	945	1	0	38	51	27	12	1093
5	3	18	256	12	2751	12	48	35	0	0	3135
6	22	16	39	0	2	546	5	8	31	83	752
7	2	6	10	1	12	14	1132	44	6	13	1240
8	1	1	0	204	37	52	0	1439	2	0	1736
9	3	12	2	62	14	6	0	45	1968	0	2033
10	14	2	0	16	83	12	0	26	0	2382	2535
	OA = 93.4%; KC = 87.5%										

## Data Availability

The data are available in the GitHub repository: https://github.com/paulinelemenkova/GRASS_ML_Djibouti, accessed on 12 June 2025.

## Abbreviations

The following abbreviations are used in this manuscript:

API	Application Programming Interface
CNN	Convolutional Neural Network
EO	Earth Observation
FAO	Food and Agriculture Organisation
GEBCO	General Bathymetric Chart of the Oceans
GIS	Geographic Information System
GMT	Generic Mapping Tool
GRASS GIS	Geographic Resources Analysis Support System GIS
KC	Kappa Coefficient
LCCS	Land Cover Classification System
OLI	Operational Land Imager
ML	Machine Learning
OA	Overall Accuracy
QGIS	Quantum Geographic Information System
RF	Random Forest
RS	Remote Sensing
SVM	Support Vector Machine
TIRS	Thermal Infrared Sensor
TIFF	Tag Image File Format
USGS	United States Geological Survey
UTM	Universal Transverse Mercator
WGS84	World Geodetic System 1984
WRS	Worldwide Reference System

## Appendix A. Initial Means for Each Multispectral Band Before Data Processing

**Table A1:** Initial mean values for pixels in spectral bands assigned to target land cover types in Djibouti for the image obtained on 2 July 2015, before iterative classification process.

Class	Initial Mean Values for Multispectral Bands of Landsat: 2015						
	1-Coastal Aerosol	2-Blue	3-Green	4-Red	5-NIR	6-SWIR-1	7-SWIR-2
1	8103.97	8671.63	9562.73	9665.43	10,102	10,216.9	9598.71
2	8421.69	9028.36	10,061.5	10,389.8	10,972.8	11,250.5	10,522.6
3	8739.4	9385.08	10,560.2	11,114.2	11,843.6	12,284.2	11,446.6
4	9057.11	9741.81	11,059	11,838.6	12,714.5	13,317.8	12,370.5
5	9374.82	10,098.5	11,557.7	12,563	13,585.3	14,351.5	13,294.4
6	9692.54	10,455.3	12,056.4	13,287.4	14,456.2	15,385.1	14,218.3
7	10,010.2	10,812	12,555.2	14,011.8	15,327	16,418.8	15,142.3
8	10,328	11,168.7	13,053.9	14,736.2	16,197.9	17,452.4	16,066.2
9	10,645.7	11,525.4	13,552.7	15,460.6	17,068.7	18,486.1	16,990.1
10	10,963.4	11,882.1	14,051.4	16,185	17,939.6	19,519.7	17,914

**Table A2:** Initial mean values for pixels in spectral bands assigned to target land cover types in Djibouti for the image obtained on 23 July 2017, before iterative classification process.

Class	Initial Mean Values for Multispectral Bands of Landsat: 2017						
	1-Coastal Aerosol	2-Blue	3-Green	4-Red	5-NIR	6-SWIR-1	7-SWIR-2
1	8074.96	8660.92	9599.33	9725.17	10,161.2	10,291.9	9670.42
2	8390.09	9009.56	10,079.2	10,419.5	11,011.6	11,300.7	10,555.8
3	8705.22	9358.2	10559.1	11,113.9	11,861.9	12,309.4	11441.2
4	9020.35	9706.85	11,039	11,808.3	12,712.3	13,318.1	12,326.7
5	9335.49	10,055.5	11,518.9	12,502.6	13,562.7	14,326.9	13,212.1
6	9650.62	10,404.1	11,998.8	13,197	14,413.1	15,335.6	14,097.5
7	9965.75	10,752.8	12,478.7	13,891.4	15,263.5	16,344.3	14,982.9
8	10,280.9	11,101.4	12,958.6	14,585.7	16,113.8	17,353.1	15,868.3
9	10,596	11,450.1	13,438.5	15,280.1	16,964.2	18,361.8	16,753.7
10	10,911.1	11,798.7	13,918.4	15,974.4	17,814.6	19,370.5	17,639.1

**Table A3:** Initial mean values for pixels in spectral bands assigned to target land cover types in Djibouti for the image obtained on 19 August 2021, before iterative classification process.

Class	Initial Mean Values for Multispectral Bands of Landsat: 2021						
	1-Coastal Aerosol	2-Blue	3-Green	4-Red	5-NIR	6-SWIR-1	7-SWIR-2
1	8317.6	8806.66	9565.01	9718.97	10,254	10,232.6	9622.06
2	8578.74	9106.8	10,005.6	10,359.3	11,061	11,207.5	10,480.5
3	8839.87	9406.95	10,446.1	10,999.7	11,867.9	12,182.4	11,338.9
4	9101	9707.09	10,886.7	11,640.1	12,674.8	13,157.4	12,197.4
5	9362.13	10,007.2	11,327.3	12,280.4	13,481.8	14,132.3	13,055.8
6	9623.26	10,307.4	11,767.8	12,920.8	14,288.7	15,107.2	13,914.3
7	9884.39	10,607.5	12,208.4	13,561.1	15,095.7	16,082.1	14,772.7
8	10,145.5	10,907.7	12,649	14,201.5	15,902.6	17,057.1	15,631.2
9	10,406.7	11,207.8	13,089.5	14,841.9	16,709.6	18,032	16,489.6
10	10,667.8	11,507.9	13,530.1	15,482.2	17,516.5	19,006.9	17,348

**Table A4:** Initial mean values for pixels in spectral bands assigned to target land cover types in Djibouti for the image obtained on 16 July 2023, before iterative classification process.

Class	Initial Mean Values for Multispectral Bands of Landsat: 2023						
	1-Coastal Aerosol	2-Blue	3-Green	4-Red	5-NIR	6-SWIR-1	7-SWIR-2
1	8504.75	9131.19	9993.94	10,161.4	10,600.6	10,554.5	9880.58
2	8747.11	9404.01	10,394.1	10,760.9	11,378.1	11,500.1	10,685.6
3	8989.47	9676.83	10,794.3	11,360.4	12,155.5	12,445.8	11,490.6
4	9231.83	9949.65	11,194.5	11,960	12,933	13,391.4	12,295.6
5	9474.19	10,222.5	11,594.7	12,559.5	13,710.4	14,337	13,100.6
6	9716.55	10,495.3	11,994.9	13,159.1	14,487.8	15,282.7	13,905.5
7	9958.91	10,768.1	12,395.1	13,758.6	15,265.3	16,228.3	14,710.5
8	10,201.3	11,040.9	12,795.3	14,358.1	16,042.7	17,173.9	15,515.5
9	10,443.6	11,313.7	13,195.5	14,957.7	16,820.1	18,119.6	16,320.5
10	10,686	11,586.6	13,595.7	15,557.2	17,597.6	19,065.2	17,125.5

## Appendix B. Iterative Cycles of Image Classification

**Table A5:** Iterative process of pixel assignment to target classes during image classification: 2015.

2015	Pixel Distribution by Categories of Land Cover Classes: 2015										
	Stable Points	1	2	3	4	5	6	7	8	9	10
Iteration 1	92.24%	1672	112	50	559	1110	860	613	390	635	929
Iteration 2	93.78%	1568	213	75	584	1057	850	622	494	647	820
Iteration 3	96.28%	1538	242	97	596	1026	843	658	541	626	763
Iteration 4	96.78%	1531	249	117	614	994	840	691	575	599	720
Iteration 5	96.97%	1531	249	137	622	980	836	714	610	568	683
Iteration 6	97.10%	1531	249	166	626	963	833	726	643	537	656
Iteration 7	97.13%	1531	250	194	632	945	834	732	669	512	631
Iteration 8	97.11%	1531	250	219	650	930	827	744	668	498	613
Iteration 9	97.52%	1531	250	243	658	936	819	732	674	480	607
Iteration 10	97.59%	1531	250	261	672	941	810	734	659	472	600
Iteration 11	97.75%	1531	251	279	681	954	798	727	650	462	597
Iteration 12	97.79%	1531	251	291	704	968	775	720	640	456	594
Iteration 13	97.95%	1531	251	305	729	964	768	710	629	450	593
Iteration 14	98.12%	1531	251	314	755	964	761	699	616	448	591

**Table A6:** Iterative process of pixel assignment to target classes during image classification: 2017.

2017	Pixel Distribution by Categories of Land Cover Classes: 2017										
	Stable Points	1	2	3	4	5	6	7	8	9	10
Iteration 1	91.34%	1698	165	76	574	1121	829	516	427	655	959
Iteration 2	93.52%	1584	263	117	620	1047	811	522	531	667	858
Iteration 3	95.37%	1518	316	137	669	996	788	553	605	640	798
Iteration 4	96.70%	1486	341	140	699	977	776	578	668	605	750
Iteration 5	97.15%	1468	357	134	718	972	771	611	707	575	707
Iteration 6	97.28%	1461	360	131	729	975	783	641	712	561	667
Iteration 7	97.51%	1456	362	123	741	986	798	651	730	528	645
Iteration 8	97.62%	1453	363	118	745	1016	795	670	725	510	625
Iteration 9	98.15%	1452	361	120	746	1031	813	672	714	502	609

**Table A7:** Iterative process of pixels’ assignment to target classes during image classification: 2021.

2021	Pixels’ Distribution by Categories of Land Cover Classes: 2021										
	Stable Points	1	2	3	4	5	6	7	8	9	10
Iteration 1	91.92%	1682	142	128	663	1062	784	485	371	626	988
Iteration 2	90.90%	1385	431	174	664	1017	766	497	467	659	871
Iteration 3	91.33%	1023	791	193	673	989	758	516	549	634	805
Iteration 4	93.64%	785	1029	198	684	985	752	540	587	621	750
Iteration 5	95.74%	666	1147	203	690	984	761	557	614	596	713
Iteration 6	96.90%	612	1201	208	694	984	771	573	641	556	691
Iteration 7	97.76%	596	1217	211	698	985	780	593	647	537	667
Iteration 8	97.88%	589	1224	216	701	990	795	603	645	513	655
Iteration 9	98.12%	588	1225	223	699	1007	798	608	644	495	644

**Table A8:** Iterative process of pixel assignment to target classes during image classification: 2023.

2023	Pixel Distribution by Categories of Land Cover Classes: 2023										
	Stable Points	1	2	3	4	5	6	7	8	9	10
Iteration 1	91.95%	1665	98	82	574	1139	882	567	370	632	1032
Iteration 2	91.27%	1393	367	114	611	1068	871	570	458	689	900
Iteration 3	92.44%	1134	623	138	641	1027	851	595	538	662	832
Iteration 4	94.08%	939	818	156	653	1012	837	618	588	644	776
Iteration 5	96.51%	872	885	173	661	1004	828	638	609	630	741
Iteration 6	97.26%	847	910	187	668	1003	820	662	606	625	713
Iteration 7	97.74%	842	915	200	676	996	829	663	621	606	693
Iteration 8	97.77%	842	915	216	681	996	825	671	635	583	677
Iteration 9	97.64%	842	915	237	683	994	829	666	648	569	658
Iteration 10	97.88%	842	915	250	700	983	830	666	656	558	641
Iteration 11	97.95%	842	915	264	711	982	826	667	655	558	621
Iteration 12	97.73%	842	915	282	719	982	822	668	655	557	599
Iteration 13	97.74%	842	915	303	721	980	832	664	650	549	585
Iteration 14	97.80%	842	915	332	710	980	837	672	639	545	569
Iteration 15	97.83%	842	915	352	723	966	844	672	634	533	560
Iteration 16	98.13%	842	915	367	733	965	840	672	627	533	547

## Appendix C. Computed Class Means for Land Cover Classes in the Region of Lake Assal, Djibouti, East Africa

(A1) $$\left|\begin{array}{ccccccccccc} \; & 1 & 2 & 3 & 4 & 5 & 6 & 7 & 8 & 9 & 10\\ 1 & 0 & \; & & \; & & \; & & \; & & \\ 2 & 1.5 & 0 & \; & & \; & & \; & & \; & \\ 3 & 5.2 & 3.0 & 0 & \; & & \; & & \; & & \\ 4 & 6.9 & 4.3 & 0.8 & 0 & \; & & \; & & \; & \\ 5 & 7.8 & 5.0 & 1.5 & 0.8 & 0 & \; & & \; & & \\ 6 & 7.9 & 5.3 & 2.2 & 1.7 & 0.9 & 0 & \; & & \; & \\ 7 & 6.2 & 4.5 & 2.4 & 2.0 & 1.5 & 0.8 & 0 & \; & & \\ 8 & 7.4 & 5.5 & 3.4 & 3.1 & 2.6 & 1.8 & 0.8 & 0 & \; & \\ 9 & 8.6 & 6.7 & 4.7 & 4.5 & 4.0 & 3.2 & 1.9 & 1.1 & 0 & \\ 10 & 11.0 & 8.6 & 6.5 & 6.5 & 6.0 & 5.0 & 3.3 & 2.4 & 1.2 & 0 \end{array}\right|\;\left|\begin{array}{ccccccccccc} \; & 1 & 2 & 3 & 4 & 5 & 6 & 7 & 8 & 9 & 10\\ 1 & 0 & \; & & \; & & \; & & \; & & \\ 2 & 1.3 & 0 & \; & & \; & & \; & & \; & \\ 3 & 3.0 & 1.6 & 0 & \; & & \; & & \; & & \\ 4 & 5.8 & 3.5 & 1.2 & 0 & \; & & \; & & \; & \\ 5 & 7.1 & 4.4 & 1.8 & 0.8 & 0 & \; & & \; & & \\ 6 & 7.4 & 4.9 & 2.3 & 1.6 & 0.9 & 0 & \; & & \; & \\ 7 & 7.5 & 5.2 & 2.9 & 2.4 & 1.8 & 1.0 & 0 & \; & & \\ 8 & 7.5 & 5.5 & 3.4 & 3.2 & 2.7 & 2.0 & 1.0 & 0 & \; & \\ 9 & 8.0 & 6.1 & 4.2 & 4.2 & 3.8 & 3.1 & 2.2 & 1.2 & 0 & \\ 10 & 8.8 & 7.1 & 7.1 & 5.2 & 5.3 & 5.0 & 4.3 & 3.4 & 1.1 & 0 \end{array}\right|$$

Equation (A1): Class separability matrices for classified images of Djibouti: 2015 and 2017.

(A2) $$\left|\begin{array}{ccccccccccc} \; & 1 & 2 & 3 & 4 & 5 & 6 & 7 & 8 & 9 & 10\\ 1 & 0 & \; & & \; & & \; & & \; & & \\ 2 & 1.6 & 0 & \; & & \; & & \; & & \; & \\ 3 & 4.1 & 2.5 & 0 & \; & & \; & & \; & & \\ 4 & 5.0 & 3.4 & 0.7 & 0 & \; & & \; & & \; & \\ 5 & 6.3 & 4.6 & 1.4 & 0.7 & 0 & \; & & \; & & \\ 6 & 6.5 & 4.9 & 2.0 & 1.4 & 0.8 & 0 & \; & & \; & \\ 7 & 5.2 & 4.1 & 2.1 & 1.7 & 1.3 & 0.7 & 0 & \; & & \\ 8 & 6.9 & 5.6 & 3.3 & 2.9 & 2.5 & 1.8 & 0.8 & 0 & \; & \\ 9 & 5.7 & 4.9 & 3.2 & 2.9 & 2.7 & 2.2 & 1.4 & 0.9 & 0 & \\ 10 & 8.4 & 7.2 & 5.2 & 4.9 & 4.8 & 4.0 & 2.7 & 2.1 & 0.9 & \end{array}\right|\;\left|\begin{array}{ccccccccccc} \; & 1 & 2 & 3 & 4 & 5 & 6 & 7 & 8 & 9 & 10\\ 1 & 0 & \; & & \; & & \; & & \; & & \\ 2 & 1.4 & 0 & \; & & \; & & \; & & \; & \\ 3 & 4.6 & 3.5 & 0 & \; & & \; & & \; & & \\ 4 & 6.1 & 5.0 & 0.8 & 0 & \; & & \; & & \; & \\ 5 & 6.6 & 5.4 & 1.3 & 0.9 & 0 & \; & & \; & & \\ 6 & 6.8 & 5.9 & 1.9 & 1.5 & 0.8 & 0 & \; & & \; & \\ 7 & 6.8 & 5.9 & 2.5 & 2.3 & 1.7 & 1.0 & 0 & \; & & \\ 8 & 5.5 & 4.8 & 2.6 & 2.5 & 2.0 & 1.5 & 0.8 & 0 & \; & \\ 9 & 7.6 & 6.8 & 4.2 & 4.1 & 3.6 & 3.0 & 1.9 & 0.8 & 0 & \\ 10 & 9.0 & 8.2 & 5.4 & 5.5 & 4.9 & 4.2 & 3.1 & 1.7 & 1.0 & 0 \end{array}\right|$$

Equation (A2): Class separability matrices for classified images of Djibouti: 2021 and 2023.

## Appendix D. Computed Class Means for Land Cover Classes in the Region of Lake Assal, Djibouti, East Africa

**Table A9:** Computed class means of 10 land cover types in Lake Assal, Djibouti. The computations are based on Digital Numbers (DNs) of pixels in the evaluated landscapes. Data: multispectral Landsat 8–9 OLI/TIRS images, band (1 to 7) for years 2015 to 2023.

Class	Band 1	Band 2	Band 3	Band 4	Band 5	Band 6	Band 7
2015							
1	7394.23	7878.92	8615.8	8244.47	8385.55	8786	8586.51
2	8998.3	9445.8	9981.23	9575.59	9422.92	9284.88	8887.37
3	9230.46	9913.19	11,138.2	11,958.2	12,864.5	12,862.9	11,816.5
4	9430.8	10,146.6	11,465.7	12,446	13,497.2	13,806.2	12,619
5	9547.38	10,303	11,752.4	12,912.6	14,092.7	14,641.3	13,360.3
6	9821.76	10,624.5	12,235.6	13,635.1	14,896.9	15,478.6	14,133.9
7	10,246.8	11,081.6	12,862.4	14,552	16,012.4	16,653.6	15,224.3
8	10,661.7	11,544	13,560.5	15,543.5	17,259.8	18,626.3	17,001.6
9	11,251.3	12,246.2	14,758.7	17,309	19,326.1	21,064.2	19,184.3
10	11,881.4	12,946.1	15,865.1	18,729.9	20,856	24,277.4	22,627.1
2017							
1	7371.5	7875.98	8664.69	8356.38	8565.28	8995.22	8778.1
2	8858.16	9357.22	9951.7	9564.73	9444.54	9371.12	8992.18
3	8945.89	9777.33	11,054.5	11,333.4	11,764	11,571.8	10,743
4	9321.25	10,018.9	11,324	12,242.3	13,244.4	13,468.5	12,268.8
5	9496.17	10,212.7	11,611.2	12,696.3	13,855.2	14,375.8	13,058.1
6	9715.96	10,479.6	12,049.1	13,373.4	14,635.2	15,185.6	13,795
7	10,087	10,877.3	12,605.2	14,223.3	15,670.3	16,380	14,930.2
8	10,526.8	11,378.8	13,341.7	15,248	16,971.7	18,216.1	16,582.3
9	11,127.8	12,082.7	14,519.5	16,951.3	19,046.9	20,773.9	18,771.8
10	11,728.2	12,813.2	15,676	18,406.1	20,794.3	24,015.7	22,082.7
2021							
1	7281.64	7688.52	8323.58	8019.43	8124.06	8480.67	8350.31
2	8578.45	8990.99	9497.29	9305.59	9360.4	9422.46	9107.76
3	9032.4	9610.46	10,681.6	11,350.9	12,512.9	12,392.4	11,404.3
4	9232.84	9855.5	11,063	11,913.4	13,238.9	13,353.2	12,168.5
5	9378.87	10,036.2	11,347.7	12,369.6	13,788.5	14,219	12,929
6	9563.91	10,256.7	11,715.6	12,988.2	14,561.6	15,110.5	13,758.7
7	10,037.5	10,785.2	12,429.3	13,969.7	15,629.4	16,072.7	14,651.6
8	10,332.3	11,120.3	12,974.7	14,809.1	16,765.2	17,972.9	16,439.1
9	10,934.6	11,846.4	14,132.9	16,407.4	18,587	20,167.3	18,278.5
10	11,319.5	12,358.9	15,074.5	17,683.4	20,102	23,286.8	21,439.7
2023							
1	7473.59	8079.42	8857.77	8532.88	8642.03	8940.82	8737.73
2	9179.37	9560.93	9889.56	9619.59	9502.14	9260.89	8893.31
3	9285.35	10,023	11,304.4	12,097.2	13,066.2	13,055	11,870.5
4	9260.65	9993.24	11324.5	12,294.7	13,624	14,111.4	12,725.8
5	9612.99	10,407.7	11,850	12,914.8	14,185.6	14,445.7	13,030.6
6	9612.99	10,407.7	11,850	12,914.8	14,185.6	14,445.7	13,030.6
7	10,105.5	10,948.7	12,646.3	14,221.3	15,852.4	16,551.5	14,997.2
8	10,551	11,459.3	13,401.9	15,287.1	17,177.8	18,217.3	16,436.4
9	10,830.2	11,812.4	14,092.6	16,381.5	18,713.8	20,687.4	18,410.8
10	11,191.6	12,288.7	14,890.2	17,469.4	20,034.6	23,154.3	20,893.2
